# Study protocol: a randomised controlled trial of a telephone delivered social wellbeing and engaged living (SWEL) psychological intervention for disengaged youth

**DOI:** 10.1186/s12888-019-2116-5

**Published:** 2019-05-06

**Authors:** Helen J. Stain, Amanda L. Baker, Christopher Jackson, Rhoshel Lenroot, Georgie Paulik, John Attia, Luke Wolfenden, Stoyan R. Stoyanov, Holly Devir, Leanne Hides

**Affiliations:** 1 School of Social and Health Sciences, Leeds Trinity University, Leeds, UK; 2 School of Medicine and Public Health, University of Newcastle, Newcastle, Australia; 3 Early Intervention Service, Birmingham and Solihull NHS Foundation Trust, Birmingham, UK; 4 School of Psychiatry, University of New South Wales, Sydney, Australia; 5 School of Psychology and Exercise Science, Murdoch University, Perth, Australia; 6 School of Psychology, University of Queensland, Brisbane, Australia; 7 School of Psychology, Queensland University of Technology, Brisbane, Australia; 8 Hunter Medical Research Institute, Newcastle, Australia; 9 Perth Voices Clinic, Perth, Australia

**Keywords:** Social inclusion, NEET, Remote therapy, Youth services, CBT, DBT, Behavioural activation, Befriending, Motivational interviewing, Social wellbeing

## Abstract

**Background:**

Internationally, from 12.2–23.4% of youth (aged 16–24 years) are not in employment, education or training (NEET). These disengaged youth are more likely to experience social exclusion, increased psychological distress and poor quality of life. Youth at risk of disengagement are less likely to access traditional support services, requiring development of innovative interventions.

**Methods:**

The trial is a single blind, three arm, randomised controlled trial evaluating the effectiveness of a telephone delivered psychological intervention for disengaged youth (12–25 years). Participants will be randomised to receive either (i) SWEL, (ii) Befriending, or (iii) Single Session Psycho-Education. Therapy will be over an 8 week period with a minimum of four and maximum of eight sessions for the SWEL or Befriending conditions, or a single session for the Psycho-Education condition. Outcomes will be assessed at baseline and at 2, 8 and 14-month follow-up with the primary outcome being re-engagement in education, training or employment.

**Discussion:**

This large, multi-site, randomised controlled trial will inform the delivery of services for young people at risk of disengaging from education or training. The provision of psychological therapy by telephone increases access by youth – especially those in rural and remote areas - both to the trial and the treatment, if adopted by services. The outcomes of this trial could have meaningful societal impact for a vulnerable population. It is expected that recruitment, intervention and retention will present challenges for the trial given the focus on disengaged youth.

**Trial registration:**

ANZCTR, ACTRN12614001212640, Registered 18 Nov 2014. Retrospectively registered.

**Ethics and dissemination:**

Ethics approval has been obtained from the participating institutions. Results of the trial will be submitted for publication in peer reviewed journals and findings presented at scientific conferences and to key service providers and policy makers.

## Background

Young people with low educational attainment and/ or limited employment are more likely to experience social exclusion [1], increased psychological distress [2] and poor quality of life [3]. Currently, global youth unemployment reaches 13.1%, three times that of adult rates [4] equating to nearly 75 million. Rates of youth (aged 16–24 years) not in employment, education or training (NEET) are reported at 23.4% in the European Union, 22.2% in the United Kingdom (UK), 15.5% in United States of America, and 12.2% in Australia. Social exclusion, such as homelessness and unemployment, are associated with risk for mental health problems [2–5]. Australian Aboriginal youth continue to have poorer educational outcomes [6] and lower levels of social and emotional wellbeing compared to non-Aboriginal youth [7–9].

Cohort studies have shown that NEET youth are more likely to have mental health problems currently, or in childhood or adolescence, compared to non NEET youth [10], and that a history of mental health problems from childhood to young adulthood is associated with increased risk for NEET at age 19 [11]. The 2010 Australian National Strategy for Young Australians prioritises improving youth health and wellbeing by empowering young people in shaping their futures by securing education, increasing family support, encouraging community participation and providing early intervention [12]. Our clinical trial aims to improve young people’s ability to make wise life choices such as participating in education.

The focus of the social well-being and engaged living intervention (SWEL) is on social engagement as a construct determined by social self-efficacy and interpersonal effectiveness. Social self-efficacy is the perceived ability of one’s self to form and maintain social relationships [13], contributing to one’s sense of agency or mastery [14]. According to Bandura [15], people with high self-efficacy are more likely to produce their own future rather than simply foretell it. This belief in the ability to alter one’s own life circumstances is fundamental to our intervention. Results of a path analysis (*N* = 664 adolescents) showed social self-efficacy beliefs influenced an adolescent’s expectations for the future, sense of self, life satisfaction and positive emotions [13]. Self-efficacy beliefs have a direct positive impact on academic and social outcomes for adolescents through an enhanced sense of mastery [16] and have been associated with decreased likelihood of dropping out of school [17]. Interpersonal effectiveness can be measured by level and range of social activity, quality of relationships (independent of school or work engagement) and life satisfaction. SWEL has been specifically designed for this trial by integrating several evidence-based Cognitive Behavioral Therapy (CBT) and Dialectical Behaviour Therapy-derived therapies in order to target sense of self, interpersonal effectiveness and affect regulation. These abilities represent key developmental challenges for adolescents [16].

CBT has a strong evidence base across a range of ages and clinical disorders [18, 19]. Its delivery by internet, videoconference or telephone has the potential to increase access to psychological interventions, particularly for people with limited mobility (e.g. youth without their own transport) and rural residents. Trials of internet-delivered CBT have had mixed results. A review of online CBT interventions for anxiety or depression found that 23 out of 26 trials had clinically significant outcomes, with effect sizes of 0.42–0.65 for depression and 0.29–1.74 for anxiety [20]. A review of 20 clinical trials for anxiety and depression found that computerised CBT was as effective as therapist led CBT and more effective than treatment as usual (TAU) [21]. However another review of 12 randomised controlled trials (RCT) for anxiety and depression found large effect sizes for therapist supported online CBT compared to small effect sizes for online CBT alone [22]. Clinicians have reported the lack of therapist contact in internet delivered CBT for children and adolescents as concerning [23].

Telephone delivered CBT ensures therapist contact and flexibility with the intervention as the therapist can tailor the session according to the young person’s presentation on the day. A meta-review of 13 remotely delivered RCTs for anxiety and depression included 10 trials of telephone-delivered psychotherapy and noted effect sizes consistent with published face to face trials [24]. A recent pilot study of telephone-delivered CBT for 10 adolescents with OCD found significant improvements in symptoms [25]. Feedback from the adolescents showed telephone CBT to be highly acceptable, convenient (less travel time), flexible (they could be away from home) and less stressful than attending a clinic [25]. Our research has shown similar results for psychological assessments conducted by videoconference compared to face to face [26].

## Objectives of the study

This trial will determine the efficacy of a telephone delivered SWEL psychological intervention for improving the social engagement and emotional wellbeing of disengaged youth. It is hypothesised that participants receiving the SWEL intervention will achieve significantly higher levels of re-engagement in education, training or employment, than those receiving Befriending, or Single Session Psycho-Education. It is also hypothesised that mental wellbeing, self-esteem, social and occupational functioning, and affect regulation will be improved for participants receiving SWEL, while mental ill-health, alcohol and other drug use will be reduced.

## Methods

### Study design

**Fig. 1:**
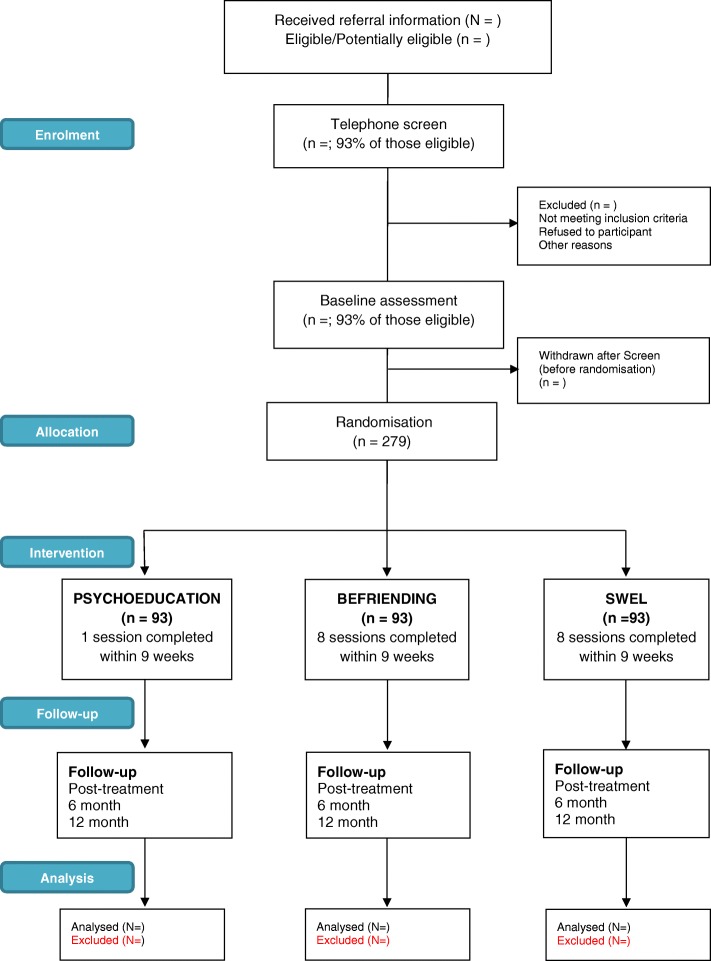
Consort diagram

The trial is a single blind three arm randomised controlled trial evaluating the effectiveness of a telephone-delivered social well-being and engaged living (SWEL) intervention for improving the vocational, social and emotional functioning of disengaged youth. Participants will be randomised to receive either (i) SWEL, (ii) Befriending, or (iii) Single Session Psycho-Education. Befriending has been applied in a RCT of CBT for youth with a first episode of psychosis [27] and has been developed in earlier clinical trials to address the nonspecific elements of psychotherapy [28]. Outcomes will be assessed at baseline and at 2, 8 and 14-month follow-up, with assessments conducted over the phone and online. Figure 1 provides an overview of the trial participant flow. Design and implementation is being carried out with the ongoing direct input from our Aboriginal communities.

### Eligibility criteria

Eligible participants will be aged 12–25 years, and at risk of disengagement from education, training or employment, defined as non-attendance for at least 28 days in the past 3 months. Given our early intervention focus, young people will be excluded if they are chronically disengaged (> 6 months). This is consistent with the core or sustained (long term disengagement) subcategory for NEET young people in the UK [29]. Exclusions will include: severe intellectual disability and/or high dependence on medical care that may impede ability to re-engage with education, training or work; diagnosed psychotic disorder; hearing impairment; or insufficient English fluency to give informed consent. Those identified as being at high risk of suicide will be excluded until suicide risk is addressed. Thereafter participants will be invited to be rescreened for eligibility.

### Recruitment and follow up procedures

Participants will be recruited through youth employment, educational and vocational training services in two Australian States: Queensland and New South Wales. These include three YMCA Vocational Schools in Brisbane and Ipswich, Yourtown (formerly Boystown), several secondary schools in Newcastle and Toowoomba Youth Service. Research team members will regularly visit each site to explain the study to staff and young people, attend the first week of vocational group programs and parent/teacher interviews, and regularly phone/email the sites to check for referrals. Parents/guardians of participants aged under 18 years will be provided a copy of the parent/guardian information statement and will be asked to confirm that the young person is capable of understanding the study and of consenting to take part. Thereafter, parents/guardians will be able to return the signed consent form, or to verbally consent over the phone to the recruitment team. The requirement for parent/guardian consent will not be enforced if the young person is considered to be a ‘mature minor’ that lives/functions independently of their parent/guardian or does not wish their parent/guardian to be involved as this may cause psychological distress or psychosocial harm. The research psychologist will determine if the young person is of sufficient age and maturity to provide informed consent based on their ability to understand and recall the aims, potential risks and benefits of the research. An Aboriginal Youth Officer will facilitate the referral of Aboriginal youth to the project. Sites will complete a referral form or provide contact details. Self-referrals will also be accepted. Recruitment will also be conducted via Facebook and an age-targeted advertisement on the university’s online research page. By clicking on the advertisement, young people will be taken to an online participant information sheet and self-referral form, submittable to the research team.

SWEL research clinical psychologists will contact new participants within 24 h of receiving referrals. They will provide detailed information about the trial, obtain consent verbal and confirm eligibility. A 20–30 min baseline assessment will be conducted by phone immediately after screening. During this assessment participant age, gender, ethnicity and location will be recorded. Thereafter participants will complete online versions of self-report measures. Reporting will follow the CONSORT guidelines (Fig. 1) [30]. Follow-up assessments consisting of an online survey and telephone interview will be conducted at 2, 8, and 14 months post-baseline by research officers blinded to treatment allocation. To enhance retention, participants will be emailed a link to the online survey 1 day before, then one and 3 days after the follow-up is due. In order to ensure that participants have received the survey link, it will also be sent via a SMS 2 days after the online survey is due. Participants will receive $AUD40 cash for each assessment time point (total $AUD160) to encourage engagement and follow-up completion. Those who have not responded to the initial calls will be contacted on a weekly basis by phone and SMS for up to 4 weeks after follow-up is due.

### Randomisation

Randomisation will be performed after the baseline survey by an automated web-based research trials management system. Participant allocation will be concealed in a secure database within the program, accessible only by the website manager. It will be generated with permuted blocks of varying sizes and stratified by age (12–18 or 19–25 years old), gender, zone (rural or urban) and indigenous status (yes or no), to allocate participants to one of the three treatment groups.

### Measures

Outcome measures are limited to 30 min for completion to minimise assessment fatigue and are completed online or by telephone. The assessment schedule is provided in Table 1.

**Table 1:** Assessment Schedule

Instrument	Domain	BL	2 m	8 m	14 m
Engagement: a Brief Time Use Questionnaire	Vocational engagement	✓			
Psychosis Screener	Psychosis	✓			
MINI Suicidality Scale	Suicidality	✓			
Demographics Accommodation, Financial support	Demographics	✓	✓	✓	✓
Engagement: a Comprehensive Time-Use Questionnaire	Vocational engagement	✓	✓	✓	✓
Multidimensional Adolescent Functioning Scale (MAFS)	Functioning	✓	✓	✓	✓
Social & Occupational Functioning Assessment Scale (SOFAS)	Social and Occupational Functioning	✓	✓	✓	✓
Global Functioning Social & Global Functioning Role Scales	Social and Role Functioning	✓	✓	✓	✓
Social Functioning Scale of the Lehman Quality of Life Scale	Social activity, quality of life, relationship quality	✓	✓	✓	✓
Mental Health Continuum Short Form	Mental wellbeing	✓	✓	✓	✓
Kessler 10 (K10)	Mental ill-health	✓	✓	✓	✓
Brief Resilience Scale	Resilience	✓	✓	✓	✓
Regulation of Emotions Questionnaire	Emotional wellbeing	✓	✓	✓	✓
WHO-ASSIST	Substance use	✓	✓	✓	✓
Perceived Empathic and Social Efficacy Scale	Social engagement	✓	✓	✓	✓
Rosenberg Self Esteem Scale	Self esteem	✓	✓	✓	✓
Treatment Recording Form	Other treatment received		✓	✓	✓
Youth Therapy Satisfaction Questionnaire	Treatment satisfaction		✓		

#### Screening measures

##### Engagement screener

The 2-item Brief Time Use Questionnaire was developed for this study to measure level of engagement in education, training, employment and job seeking. Participants report the date they last attended school, work or training, and the number of attendance days over the past 6 months to assess inclusion criteria for recent disengagement. They report the number of attendance days for each month, going backwards from the day of the survey (excluding school holidays). A ratio of engagement is calculated as a percentage based on actual days attended/days required to attend. This allows for a correct calculation of engagement, accounting for different study or work schedule requirements. If a participant’s ratio of attendance is less than two thirds in the last 3 months (i.e. they missed at least one third of the days of school/work/training in the last 3 months) then they are considered ‘disengaged’ and eligible for the program. If a total of zero days of attendance are reported over the past 6 months, participants are considered ‘too disengaged’ and excluded from the study. If more than two thirds of the days of attendance is reported over the past 3 months then participants are also excluded as they are considered ‘too engaged’.

##### Active psychosis

The 7-item Psychosis Screener from the Composite International Diagnostic Interview (CIDI) will be used to assess for the presence of characteristic psychotic symptoms [31].

##### Suicidal ideation

The 6-item Suicidality Scale of the Mini International Neuropsychiatric Interview (MINI) will be used to assess suicide risk [32].

Based on eligibility, a skip-logic implemented at the end of the screening survey leads directly into the Baseline survey, or to the end of the survey.

#### Baseline and follow-up measures

##### Demographics

Age, gender, ethnicity, living arrangements, financial support, years of education and relationship status.

##### Engagement

A Comprehensive Time-Use Questionnaire of 28 items with skip logic measures the level of engagement in education, training, employment and seeking work since the last follow-up. Items include type of education attended (e.g. school, certificate, etc.), number of hours attended and length of enrolment in the program. The items for training, employment, job searching and volunteer work follow the same pattern.

##### Functioning

The Multidimensional Adolescent Functioning Scale (MAFS) is a 23-item self-report measure of general, family and peer functioning [33]. Social and occupational functioning is measured using the Social and Occupational Functioning Assessment Scale (SOFAS) [34] and the Global Functioning: Social (GF-Social) and Global Functioning: Role (GF-Role) scales [35, 36]. Social activity, quality of life and relationship quality are measured by the Social Functioning Scale (SFS) of the Lehman Quality of Life Scale [37].

##### Mental ill-health and wellbeing

Self-report information on the young person’s mental health history is collected including current mental health treatment, and family history of mental health problems. The Mental Health Continuum Short Form is a 14-item positive mental health scale measuring emotional, psychological and social wellbeing [38]. The Kessler 10 (K10) [39] is a 10-item self-report questionnaire measuring psychological distress in the past month. Normative data indicates a cut-off of ≥17 is at the 75th percentile among Australian youth [40]. The young person’s ability to bounce back or recover from stress is measured by the 6-item Brief Resilience Scale [41].

##### Affect regulation

The Regulation of Emotions Questionnaire [42] (21 items) was developed and tested in a sample of adolescents measuring adaptive and maladaptive strategies for processing emotions.

##### Alcohol and other drug use

The 8-item Alcohol, Smoking and Substance Involvement Screening Test Version 3.0 (ASSIST) [43] measures lifetime and recent (past 3 months) use of 10 substances, as well as abuse and dependence symptoms. It was developed by the World Health Organisation (WHO) as a screening instrument for all psychoactive substances, with high levels of internal consistency, construct, concurrent and discriminant validity [44].

##### Self-efficacy

The Perceived Empathic and Social Efficacy Scale is an 11-item measure that assesses self-efficacy beliefs regarding both empathic responding to others’ needs or feelings, and managing interpersonal relationships [45]. The Rosenberg Self-Esteem Scale is a 10-item measure of global self-esteem [46].

### Psychological interventions

Research therapists will complete a one-day training workshop [led by HS and/or LH] for the SWEL, Befriending and Single Session Psycho-Education conditions. An Aboriginal Youth Worker will be available to support the research therapists and the Aboriginal participants with treatment engagement, attendance and delivery. All interventions will be delivered by telephone and participants will be reimbursed $AUD10 cash per session to maximise retention and treatment completion. Participants in the SWEL and Befriending conditions will initially be encouraged to participate in at least four sessions. Once engaged they will be offered four additional sessions, receiving a maximum of eight (one per week). Participants randomised to Psycho-Education will receive one session over the eight-week period. Sessions are 30–60 min duration and will be audio-recorded. Therapists will ensure participants are in an appropriate setting during therapy calls to maintain participant privacy and confidentiality.

#### SWEL intervention

The SWEL manualised intervention is designed to enhance young people’s vocational engagement by targeting their emotional and social self-efficacy. It has a CBT framework and is divided into two sets of modules. The core modules are delivered to all participants in four sessions. Thereafter, participants are offered four further sessions on a needs basis, to revisit core modules and/or add optional modules. The four core modules are assessment, formulation/goal setting, emotion regulation/interpersonal effectiveness, and behavioural activation/engaged living. The three optional modules are self-esteem, problem solving, and drug and alcohol use using motivational interviewing. The final session serves for review and consolidation of skills.

#### Befriending intervention

Befriending is a manualised intervention developed in earlier clinical trials to address the non-specific elements of psychotherapy [47]. It controls for therapist contact time, client expectancies, therapeutic alliance and therapist factors (e.g., warmth and understanding) and has been used in a randomised controlled trial of CBT for youth with a first episode of psychosis [28]. It does not apply therapeutic techniques specific to major models of psychotherapy and focuses on everyday events and topics using a conversational, friendly approach, without problem solving or examination of emotions. Befriending will be delivered for up to eight weekly sessions to allow for the same level of contact as the SWEL condition.

#### Single session psycho-education plus TAU intervention

This single-session intervention was specifically developed for this study to deliver feedback from the baseline assessment and information on relevant topics for disengaged youth including wellbeing, depression, anxiety, anger and substance use. The session will be delivered using a conversational approach.

### Treatment satisfaction and fidelity

Treatment satisfaction will be assessed at 2-month follow up using the 7-item Youth Therapy Satisfaction Questionnaire. This adaptation of the Youth Satisfaction Questionnaire [48] includes two additional questions asking if the participant would recommend telephone counselling to a friend and if they prefer telephone to face to face counselling. All treatment sessions will be audio-recorded and session component checklists will be completed following each session for controlling the content and dose of therapy each participant receives. A random sample (20% of participants; evenly spread across the three treatment groups) of session recordings will be independently rated for treatment fidelity using an adapted version of the ACE Treatment Integrity Measure (ATIM) [49]. Every treatment session delivered to the 20% of participants selected will be rated. The session checklists will also be completed to confirm the content and dose of treatment delivered.

#### Adaptation of the ATIM [49]

The ATIM was chosen to measure treatment integrity as it was designed to detect adherence and differentiation between CBT and Befriending. Since CBT treatments differ in their emphasis on specific CBT techniques, it is important that treatment fidelity measures be tailored to the treatment manual [50, 51]. Consequently, the ATIM was adapted to the specific treatment manuals used in the present study. This resulted in the addition of five SWEL strategy items (*Therapist provides assessment feedback; Therapist works with client to identify client’s strengths/positive qualities; Therapist reviews and summarises session or recaps on previous session; Therapist guides client in Behavioural Activation; Therapist introduces session/sets agenda*), one befriending strategy item (*Client and therapist engage in neutral conversations about day to day topics*), one general therapy technique item (*Therapist does suicide risk assessment*) and one item to assess differentiation between three interventions rather than two (i.e., CBT, Befriending and Psycho-Education). Three items were also removed because they involved strategies not used in the present study (*Therapist and client work on thought records; Therapist and client engage in role reversal exercises; Therapist engages client in exposure training*).

A competence measure was also added to the ATIM as this is a key component of treatment integrity [50–52]. For each strategy endorsed as present in each session, the following therapist competence item was added, “How competent was the therapist at delivering this component” rated on a 7-point Likert scale *(Very Poor to Excellent*). The item was based on competence items used in the established Yale Adherence and Competence Scale [53]. The final 69-item measure consists of 34 items for assessment of adherence, 34 for competence, and one item asking the rater to guess which therapy was delivered. Six of the adherence items target Befriending, 23 target SWEL and five target general therapy techniques (e.g., *collaboration, empathy, professionalism*).

### Assessment integrity

Baseline and follow up assessments will be audio recorded. Reliability and adherence to protocol will be assessed by an independent researcher on a random sample of 20% of participants. Research officers will receive fortnightly supervision to monitor assessment, retention and blinding. The chief investigators, follow-up assessor and trial statistician will be blind to treatment group allocation.

### Sample size calculation

A total of 279 youth (93 individuals per group) will be recruited. Allowing for a 30% loss to follow-up, the trial will have 80% power and a 5% type I error to find a Cohen’s *d* of 0.5, i.e. moderate effect size. The significance level is adjusted by a factor of 2 to control the overall type I error rate at 5% for the 2 pair-wise comparisons required to test the primary hypotheses. This estimate is conservative and does not take into account the added power from adjusting for an individual’s measure at baseline or the repeated measures of the outcomes.

### Statistical analysis

The primary outcome of this study will be engagement in education, training or employment (Time-Use Questionnaire) at the 2 month time point. Secondary outcomes include the Time-Use Questionnaire at 8 and 14 months, as well as social activity, quality of life and relationship quality (SFS), perceived social self-efficacy (Perceived Empathic and Social Efficacy Scale), sense of self (Rosenberg Self-Esteem Scale), and functioning (SOFAS, GF: Social, GF- Role, SFS, MAFS), measured at baseline, end of treatment (2 months) and at 6 and 12 months post treatment.

The primary hypothesis is that the overall engagement score (calculated as actual/expected engagement in school/work/training) of youth who are randomised to active treatment (SWEL) will be higher than those randomised to either Befriending or Psycho-Education at the 2 month time point. Differences between treatment groups will be tested within a Linear Mixed Model (LMM). The outcome in the model will be individual total engagement scores at each post treatment time point and the predictor variables in the model will include treatment group, time, the interaction between treatment group and time, baseline engagement score and the stratifying variables. The model will include a random intercept term to control for the repeated measurements on individuals. All analyses will be based on the intention to treat principle. Differences between groups in all secondary outcomes will be tested using the same approach as outlined for the primary outcome variable. Additional LMM will be used to test for a difference between treatment groups in the trend of each of these outcomes from baseline to 12 months post-treatment using data from all time points. Subgroup analyses will be conducted to examine the similarity of the treatment effect across age groups.

### Trial duration

July 2014 – June 2018.

### Ethics and dissemination

Safety and risk management protocols were devised to manage safety or urgent treatment issues. Young people recruited to this study and subsequently identified as needing more intensive treatment will be appropriately referred. A clinical committee comprised of LH, AB, the project manager, research clinicians and follow-up researchers will meet fortnightly to resolve treatment difficulties and discuss any departures from the protocol. The authors will meet quarterly to monitor the study’s implementation, clinical and research integrity. The informed consent of young people will be obtained by recruitment staff and parental or guardian consent for people aged 12–15 years will be sought.

## Discussion

This study protocol describes a large, multi-site, randomised controlled trial of a telephone-delivered psychological intervention for improving the social engagement and emotional wellbeing of young people who are disengaging from education, employment or training. The engagement of young people in completion of education or training is a major economic and societal concern in many countries. Internationally, many governments have distributed short term funding to non-government organisations in an effort to address this issue. However, there is a lack of an evidence base to guide interventions for disengaged youth and the intermittent nature of funding for non-government organisations challenges the sustainability of services.

The SWEL study aims to determine the effectiveness of a telephone delivered psychological intervention in improving the engagement of young people in education, employment or training. The provision of psychological therapy by telephone increases access by youth – especially those in rural and remote areas - both to the trial and the treatment, if adopted by services. It is hypothesised that young people receiving the SWEL intervention will achieve significantly higher levels of reengagement in education, training or employment, than those receiving Befriending, or Single Session Psycho-Education. It is also hypothesised that mental wellbeing, self-esteem, social and occupational functioning, and affect regulation will be improved for participants receiving SWEL, while mental ill-health, alcohol and other drug use will be reduced. The outcomes of this trial could have meaningful societal implications for a vulnerable population. Recruitment to the trial was delayed following the loss of national funding to the referral services but commenced in July 2014 and will end in June 2018.
